# Clinical features of bacterial meningitis among hospitalised children in Kenya

**DOI:** 10.1186/s12916-021-01998-3

**Published:** 2021-06-04

**Authors:** Christina W. Obiero, Neema Mturi, Salim Mwarumba, Moses Ngari, Charles R. Newton, Michaël Boele van Hensbroek, James A. Berkley

**Affiliations:** 1 Clinical Research Department, KEMRI-Wellcome Trust Research Programme, P.O. Box 230 80108, Kilifi, Kenya; 2 Department of Global Health, Faculty of Medicine, University of Amsterdam, Amsterdam, The Netherlands; 3 Department of Microbiology, KEMRI-Wellcome Trust Research Programme, Kilifi, Kenya; 4 The Childhood Acute Illness and Nutrition (CHAIN) Network, Nairobi, Kenya; 5 Department of Psychiatry, University of Oxford, Oxford, UK; 6 Centre for Tropical Medicine & Global Health, Nuffield Department of Medicine, University of Oxford, Oxford, UK

**Keywords:** Meningitis, Children, Clinical features, Signs, Lumbar puncture, Cerebrospinal fluid, Conjugate vaccines, Low- and middle-income countries

## Abstract

**Background:**

Diagnosing bacterial meningitis is essential to optimise the type and duration of antimicrobial therapy to limit mortality and sequelae. In sub-Saharan Africa, many public hospitals lack laboratory capacity, relying on clinical features to empirically treat or not treat meningitis. We investigated whether clinical features of bacterial meningitis identified prior to the introduction of conjugate vaccines still discriminate meningitis in children aged ≥60 days.

**Methods:**

We conducted a retrospective cohort study to validate seven clinical features identified in 2002 (*KCH-2002*): bulging fontanel, neck stiffness, cyanosis, seizures outside the febrile convulsion age range, focal seizures, impaired consciousness, or fever without malaria parasitaemia and Integrated Management of Childhood Illness (IMCI) signs: neck stiffness, lethargy, impaired consciousness or seizures, and assessed at admission in discriminating bacterial meningitis after the introduction of conjugate vaccines. Children aged ≥60 days hospitalised between 2012 and 2016 at Kilifi County Hospital were included in this analysis. Meningitis was defined as positive cerebrospinal fluid (CSF) culture, organism observed on CSF microscopy, positive CSF antigen test, leukocytes ≥50/μL, or CSF to blood glucose ratio <0.1.

**Results:**

Among 12,837 admissions, 98 (0.8%) had meningitis. The presence of *KCH-2002* signs had a sensitivity of 86% (95% CI 77–92) and specificity of 38% (95% CI 37–38). Exclusion of ‘fever without malaria parasitaemia’ reduced sensitivity to 58% (95% CI 48–68) and increased specificity to 80% (95% CI 79–80). IMCI signs had a sensitivity of 80% (95% CI 70–87) and specificity of 62% (95% CI 61–63).

**Conclusions:**

A lower prevalence of bacterial meningitis and less typical signs than in 2002 meant the lower performance of *KCH-2002* signs. Clinicians and policymakers should be aware of the number of lumbar punctures (LPs) or empirical treatments needed for each case of meningitis. Establishing basic capacity for CSF analysis is essential to exclude bacterial meningitis in children with potential signs.

**Supplementary Information:**

The online version contains supplementary material available at 10.1186/s12916-021-01998-3.

## Background

Childhood bacterial meningitis is associated with significant mortality and neurocognitive sequelae [1, 2]. The disease burden is highest in low- and middle-income countries (LMICs) where a quarter of children who survive vaccine-preventable meningitis develop post-discharge complications [2, 3]. Prompt recognition and antimicrobial treatment with cerebrospinal fluid (CSF) penetration for an adequate duration are critical.

CSF culture is the gold standard for bacterial meningitis but has limited sensitivity [4] as it may be compromised by prior administration of antimicrobials [5] and is usually unavailable or unreliable in public hospitals in sub-Saharan Africa. Public hospitals also often lack adequate CSF microscopy capacity, and lumbar puncture (LP) may be commonly ordered but not done [6, 7]. Thus, antimicrobial management decisions are often based on clinical features only.

The World Health Organization (WHO) advises suspecting bacterial meningitis if one or more of the following are present: convulsions, inability to drink, irritability, lethargy, impaired consciousness, a bulging fontanel, or neck stiffness [8]. However, this recommendation is based on limited evidence collected prior to the introduction of *Haemophilus influenzae* type b (Hib) and *Streptococcus pneumoniae* conjugate vaccines targeting the leading causes of bacterial meningitis.

In the Gambia ~20 years ago, a set of Integrated Management of Childhood Illness (IMCI) signs (lethargy, impaired consciousness, convulsions, or a stiff neck) [9] had 98% sensitivity and 72% specificity in predicting bacterial meningitis [10]. Concurrently, among children aged ≥60 days at Kilifi County Hospital (KCH), Kenya, a bulging fontanel, neck stiffness, cyanosis, seizures outside the febrile convulsions age range, focal seizures, and impaired consciousness were identified as indicators of bacterial meningitis (*KCH-2002*) [11]. These findings were incorporated into Kenyan national paediatric guidelines [12].

Hib and 10-valent pneumococcal conjugate vaccines at 6, 10, and 14 weeks of age without booster were introduced in Kenya in 2001 and 2011, respectively, resulting in a markedly reduced incidence and mortality from bacterial meningitis [13–17]. Since the early 2000’s severe malaria, which may mimic bacterial meningitis [18], has declined, with changes in age and disease profile reported at several centres in Africa [19–21].

Changes in epidemiology, patient profile and differential diagnoses may have altered associations between clinical features and bacterial meningitis. We therefore performed a revalidation study of the *KCH-2002* and IMCI signs among children aged ≥60 days.

## Methods

### Location and participants

KCH is a public hospital serving a mostly rural population. Paediatric care is supported by the KEMRI/Wellcome Trust Research Programme. Children aged 60 days to 13 years hospitalised at KCH between January 1, 2012, and December 31, 2016, were included in this analysis.

### Procedures

All children admitted were systematically assessed using standardised demographic and clinical proforma by trained clinicians at admission, and data were entered on a database in real-time. All admissions had a complete blood count, malaria slide, and blood culture. LP was performed at admission if suggestive signs were present, or if a child developed new clinical features of meningitis according to the WHO [8] and Kenyan guidelines [12] detected through daily clinical reviews until discharge. LP was deferred in children with cardiorespiratory compromise or suspicion of raised intracranial pressure [22]. Children with suspected meningitis were treated empirically with penicillin plus chloramphenicol or ceftriaxone (as per national and WHO guidelines [8, 12]) while awaiting LP results. Once available, treatment was modified based on culture and susceptibility profile as needed. Data collection (SSC1433) and this analysis (SSC3001) were approved by the KEMRI Scientific and Ethics Review Unit.

### Laboratory analysis

CSF examination included leukocyte and red blood cell (RBC) count using the Neubauer counting chamber method, and if leukocyte count >10 cells/μl, differential leukocyte count, Gram and Indian ink staining, latex antigen agglutination tests (Wellcogen™ Bacterial Antigen kit for *S. pneumoniae*, *H. influenzae*, *N. meningitidis,* and CrAg Lateral Flow Assay kit Ref CR2003 for *Cryptococcus neoformans*) were done. CSF and blood samples were cultured, and pathogens identified using standard methods as previously described [11, 18]. Coagulase-negative S*taphylococci* were considered non-significant [23]. CSF protein, glucose, and concurrent blood glucose were measured on an ILab Aries analyser (Werfen, Germany). External quality assurance was by the United Kingdom External Quality Assessment Service, and Good Clinical Laboratory Practice was accredited by Qualogy, UK [11].

### Definitions

For this analysis, we used the *KCH-2002* [11] definition of bacterial meningitis: (i) positive CSF culture for a known pathogen, (ii) positive CSF antigen test, (iii) an organism observed on CSF microscopy (Gram stain or Indian Ink), (iv) CSF leucocyte count ≥50 cells/μL, or (v) CSF to blood glucose ratio <0.1. We also defined possible meningitis as CSF leucocyte count >10–49 cells/μL in the absence of the above criteria.

### Statistical analysis

For the primary analysis, children who underwent LP not meeting meningitis criteria or without an LP were classified as not having meningitis, as was assumed in *KCH-2002*. We initially excluded children with possible meningitis [11] and calculated the highest criterion for meningitis in the order given above.

We examined the performance of *KCH-2002* [11] and IMCI signs (neck stiffness, lethargy, impaired consciousness, or seizures) [9] at admission by calculating their sensitivity, specificity, positive predictive value (PPV), and negative predictive value (NPV) for meningitis diagnosed by LP either at admission or at any time during hospitalisation versus no meningitis, defined as negative CSF analysis or no clinical suspicion of meningitis until discharge from hospital. We calculated the number of LPs needed to identify one case of meningitis as the inverse of the risk difference obtained by subtracting the prevalence of meningitis in each group from that in the group without the indicators of interest. As sensitivity analyses, we (i) included possible meningitis cases, (ii) excluded those who died before LP, and (iii) used a narrow microbiological definition of meningitis (positive CSF culture for a known pathogen, positive CSF antigen test, an organism observed on CSF microscopy (Gram stain or Indian Ink), or CSF leucocyte count >10 cells/μL plus a positive blood culture).

Proportions were compared using the chi-squared test or Fisher’s exact test. Continuous variables were compared using Wilcoxon rank-sum test. All analyses used Stata version 15 (Stata Corp, USA).

## Results

There were 12,986 admissions aged 60 days to 13 years: 2975 (23%) <1 year, 6248 (48%) 1–4 years, and 3763 (29%) ≥5 years old; 463 (3.6%) were HIV antibody positive. Two thousand six hundred-two (20%) children had an LP, of which 409 (16%) were aged <1 year. LPs were more commonly done among children aged 1–5 years [1484/6248 (24%)] than in children aged >5 years [709/3,763 (19%)] or <1 year [409/2975 (14%)], *P<0.001*. A positive malaria smear was present in 1189 (46%) children who had an LP. Of 10,384 children who did not have an LP, 565 died before an LP (193 (34%) <1 year, 230 (41%) 1–5 years and 142 (25%) ≥5 years) while 9819 survived (2373 (24%) <1 year, 4534 (46%) 1–5 years, and 2912 (30%) ≥5 years) (*P<0.001*). Median [interquartile range (IQR)] age of 565 children who died before an LP was 21 months (8.0–60) compared to 20 months (9.5–59) in 88 children who died after an LP (*P=0.874*).

### Meningitis cases

**Fig. 1:**
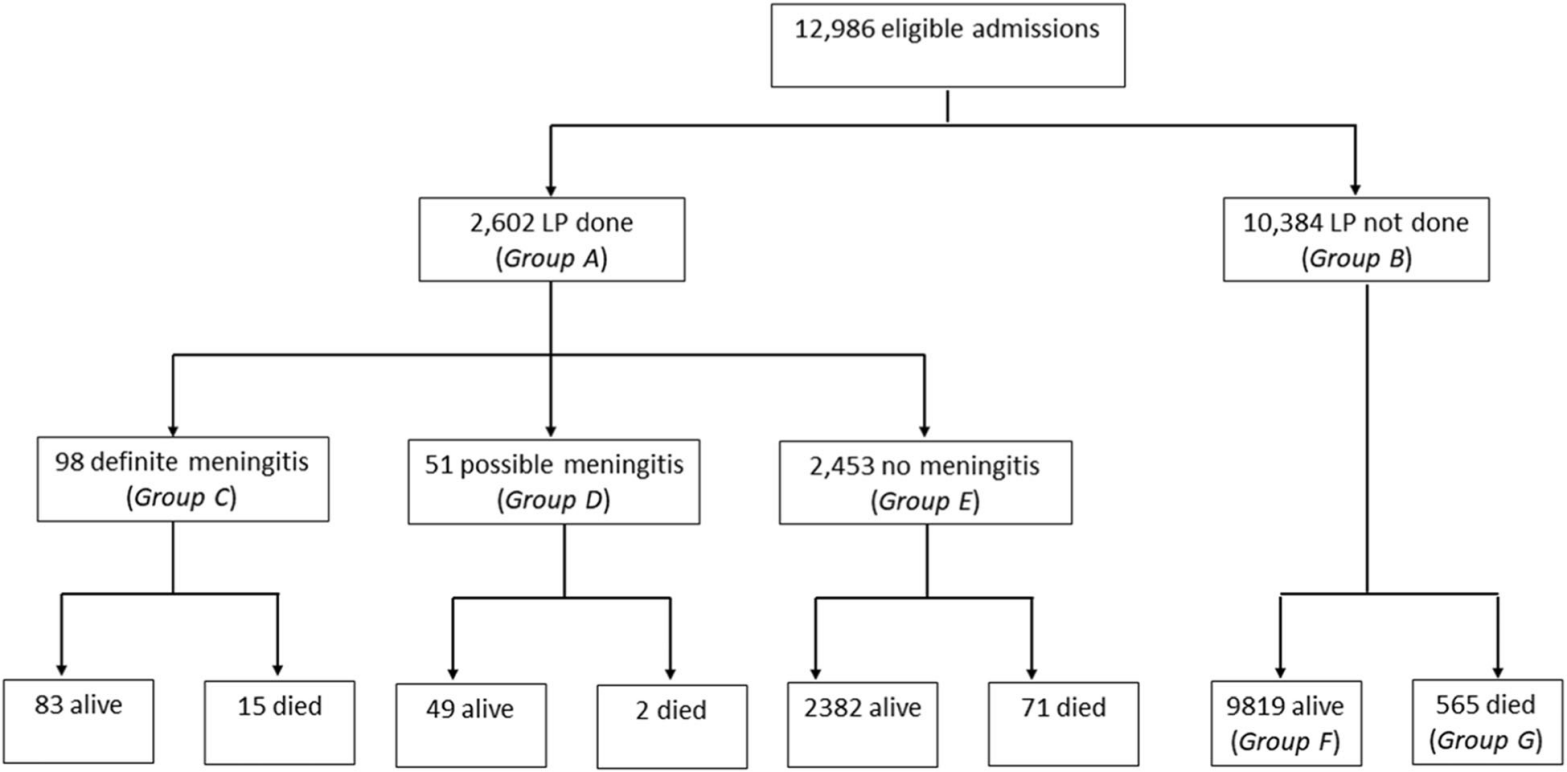
Flow chart of study participants. Abbreviation: LP, lumbar puncture

Ninety-eight children had meningitis (Fig. 1, Group C): 0.8% of 12,986 admissions and 3.8% of 2602 children with an LP. Fifty-one (0.4%) children had possible meningitis (Group D) and were excluded from the primary analysis. Median (IQR) ages of children with meningitis, possible meningitis, or no meningitis were 25 (7.4–77), 40 (11–83), and 29 (12–67) months, respectively *(P=0.167)*. Fifteen (15%) meningitis cases died during hospitalisation; 2.3% (15/653) of all inpatient deaths.

Leading CSF pathogens were *S. pneumoniae* (16 culture-positive and 4 antigen-positive) and *H. influenzae* (5 culture-positive and 3 antigen-positive) (Table 1). Fifty (51%) meningitis cases had CSF leukocyte count ≥50/μl only. One hundred twenty (4.8%) of 2521 children had differential leukocyte count done of which 118 (98%) had polymorphonuclear cell predominance (≥60%) and 77 had meningitis. Five (2.0%) of 249 children with CSF RBC count ≥500 cells/μL had positive CSF cultures while 4 (1.6%) children missed leukocyte counting due to grossly blood-stained CSF. Thirty-three (34%) children with meningitis had positive blood culture; 23 matched CSF isolates (13 *S. pneumoniae*, 4 *H. influenzae*, 2 *Salmonella* spp., and 1 each of *E. coli*, *K. pneumoniae*, *P. aeruginosa*, and *C. neoformans*). Forty-one (42%) meningitis cases had turbid CSF.

**Table 1:** Diagnostic criteria for meningitis and organisms detected

Diagnostic criteria	Age <1year (***n***=33)	Age 1–4years (***n***=35)	Age ≥5years (***n***=30)	Total positives	Highest criteria for meningitis^**a**^
**CSF culture**					
**Gram positive**					
*Streptococcus pneumoniae*	5	5	6	16^b^	16
*Haemophilus influenzae*	3	2	0	5^c^	5
*Staphylococcus aureus*	1	0	0	1^d^	1
**Gram negative**					
*Escherichia coli*	0	1	0	1	1
*Klebsiella pneumonia*	1	1	0	2^e^	2
Non-typhoidal *Salmonella* sp.	2	0	0	2^f^	2
*Pseudomonas aeruginosa*	1	0	0	1^g^	1
*Proteus mirabilis*	1	0	0	1^h^	1
*Cryptococcus neoformans*	0	0	2	2^i^	2
**Total**	**14**	**9**	**8**	**31**	**31**
**Latex antigen test**					
*Streptococcus pneumoniae*	7	5	6	18	4^j^
*Haemophilus influenzae*	2	3	0	5	3^k^
*Cryptococcus neoformans*	0	0	2	2	1
**Total**	**9**	**8**	**8**	**25**	**8**
**Gram-stain**					
Gram-positive cocci	8	8	5	21	4
Gram-negative rods	4	3	0	7	0
**Total**	**12**	**11**	**5**	**28**	**4**
**Indian ink**	**0**	**0**	**1**	**1**	**0**
**CSF WCC ≥50 cells/μl**	29	29	27	85	52^l^
**CSF/blood glucose ratio <0.1**	**7**	**5**	**3**	**15**	**3**
**Total**					**98**

^a^Hierarchical order of criteria for meningitis: (i) positive CSF culture for a known pathogen, (ii) positive CSF antigen test, (iii) an organism observed on CSF microscopy (Gram stain or Indian Ink), (iv) CSF leucocyte count ≥50 cells/μL, and (v) CSF to blood glucose ratio <0.1

^b^14/16 had WCC ≥50 cells/μl, 14/16 had positive *S. pneumoniae* antigen test, 14/16 had positive Gram stain, and 5/16 had CSF/blood glucose ratio <0.1

^c^5/5 had WCC ≥50 cells/μl, 2/5 had positive *H. influenzae* antigen test, 3/5 had positive Gram stain, and 3/5 had CSF/blood glucose ratio <0.1

^d^Had WCC ≥50 cells/μl and positive Gram stain

^e^1/2 had WCC ≥50 cells/μl and positive Gram stain

^f^1/2 had WCC ≥50 cells/μl and positive Gram stain. 1/2 had CSF/Blood glucose ratio <0.1

^g^Had WCC ≥50 cells/μl and positive Gram stain

^h^Had WCC ≥50 cells/μl, positive Gram stain and CSF/blood glucose ratio <0.1

^i^1/2 had WCC ≥50 cells/μl, positive Indian Ink stain and positive Cryptococcal antigen test

^j^2/4 had WCC ≥50 cells/μl and positive Gram stain

^k^3/3 had WCC ≥50 cells/μl, 2/3 had positive Gram stain, and 1/3 had CSF/blood glucose ratio <0.1

^l^2/52 had CSF/blood glucose ratio <0.1

### Admission clinical features

Two thousand three hundred thirty (79%), 3762 (60%), and 2007 (54%) children aged <1, 1–5, and ≥5 years, respectively, presented with *KCH-2002* signs (*P<0.001*), while 899 (30%), 2661 (43%), and 1391 (37%) had IMCI signs (*P<0.001*). Bulging fontanel, neck stiffness, impaired consciousness, seizures outside the febrile convulsion age range, focal seizures, history of fever, and axillary temperature ≥39°C were more common among children with meningitis than without, and malaria was less common (Table 2). Of 8099 children with *KCH-2002* signs, 485 (6.0%) died before LP (277 (57%) within 24 h of admission). Of 4951 children with IMCI signs, 359 (7.3%) died before LP (240 (67%) within 24 h of admission).

**Table 2:** Clinical features at admission

	No meningitis (***n***=12,837)	Meningitis (***n***=98)	***P*** value^**a**^
Bulging fontanelle^b^			
Absent	4490 (98)	37 (82)	<0.001
Present	51 (1.1)	8 (18)	
Missing	29 (0.6)	0 (0)	
Neck stiffness			
Absent	12,652 (99)	79 (81)	<0.001
Present	96 (0.8)	19 (19)	
Missing	89 (0.7)	0 (0)	
Cyanosis			
Absent	12,702 (99)	98 (100)	0.594
Present	46 (0.3)	0 (0)	
Missing	89 (0.7)	0 (0)	
History of seizures within febrile convulsion age range^c^			
No seizures	9990 (78)	48 (49)	<0.001
Seizures within febrile convulsion age range	2187 [17]	34 (35)	
Seizures outside febrile convulsion age range	573 (4.5)	16 (16)	
Missing	87 (0.7)	0 (0)	
Type of convulsion at any age			
No seizures	9990 (78)	48 (49)	<0.001
Unknown type	81 (0.6)	2 (2.0)	
Generalised^d^	2354 [18]	40 (41)	
Focal^e^	325 (2.5)	8 (8.2)	
Missing	87 (0.7)	0 (0)	
Conscious level			
Normal	9338 (73)	46 (47)	<0.001
Lethargic	1444 [11]	16 (16)	
Agitated	159 (1.2)	2 (2.0)	
Impaired consciousness^f^	1807 [14]	34 (35)	
Missing	89 (0.7)	0 (0)	
History of fever			
Absent	4070 (32)	10 (10)	<0.001
Present	8681 (68)	88 (90)	
Missing	86 (0.7)	0 (0)	
Axillary temperature, ^0^C			
<36	717 (5.6)	4 (4.1)	0.032
36-38.9	10,080 (79)	68 (69)	
≥39	2016 [16]	26 (27)	
Missing	24 (0.2)	0 (0)	
Malaria			
Negative	10,346 (81)	84 (86)	0.201
Positive	2491 [19]	14 (14)	

^a^Compares children with definite meningitis (Group B, *n*=98) to children with no meningitis [Group A (*n*=10,384) + Group D (*n*=2453)]. Excludes children with possible meningitis (Group C, *n*=51)

^b^Cut-off age for assessment of fontanel closure was 18 months. Analysis of bulging fontanel was limited to 4615 children aged ≤18 months (45 with meningitis and 4570 without meningitis); hence, column totals are less than those of other variables on the table

^c^Febrile convulsions occurring in children age between 6 months and 6 years

^d^Involving both sides of the body with associated loss of consciousness

^e^Focal, involving one side of the body, and may or may not become generalised

^f^Blantyre Coma Score (BCS) <4 up to 9 months and <5 at ≥9 months (Eyes: 1=watches/follows, 0=none; Cry: 2=normal, 1=moan/weak, 0=none; Motor: 2=localises stimulus, 1=withdraws, 0=other/none)

### Performance of clinical features

#### KCH-2002

One or more *KCH-2002* signs were present in 8099 children, of whom 84 (1.0%) had meningitis compared with 14/4836 (0.3%) without *KCH-2002* signs: sensitivity 86% (95% CI 77–92), specificity 38% (95% CI 37–38), PPV 1.0% (95% CI 0.8–1.3), and NPV 100% (95% CI 99–100). One hundred thirty-four children (95% CI 99–208) presenting with ≥1 *KCH-2002* signs would need to undergo an LP for each case of meningitis identified (Table 3).

**Table 3:** Comparison of potential screening criteria at admission for definite meningitis (excluding children with possible meningitis only (Group C, *n*=51))

Screening criteria	No. with criteria	No. with meningitis	Sensitivity % (95% CI)	Specificity % (95%CI)	PPV % (95% CI)	NPV % (95% CI)	NNLP (955% CI)
Bulging fontanel^a^ or neck stiffness	161	23	23.5 (15.5–33.1)	98.9 (98.7–99.1)	14.3 (9.3–20.7)	99.4 (99.3–99.5)	7 (5–12)
Cyanosis or any of the above	207	23	23.5 (15.5–33.1)	98.6 (98.3–98.8)	11.1 (7.2–16.2)	99.4 (99.3–99.5)	10 (7–16)
Seizures outside 6 months to 6 years or any of the above	771	33	33.7 (24.4–43.9)	94.3 (93.8–94.6)	4.3 (3.0–6.0)	99.5 (99.3–99.6)	27 (19–43)
Focal seizures or any of the above	1023	39	39.8 (30.0–50.2)	92.3 (91.9–92.8)	3.8 (2.7–5.2)	99.5 (99.4–99.6)	30 (22–47)
Impaired consciousness or any of the above	2659	57	58.2 (47.8–68.1)	79.7 (79.0–80.4)	2.1 (1.6–2.8)	99.6 (99.5–99.7)	57 (43–85)
Fever without malaria parasitemia or any of the above	8099	84	85.7 (77.2–92.0)	37.6 (36.7–38.4)	1.0 (0.8–1.3)	99.7 (99.5–99.8)	134 (99–208)
IMCI referral criteria: neck stiffness, lethargy, impaired consciousness, or seizures	4951	78	79.6 (70.3–87.1)	62.0 (61.2–62.9)	1.6 (1.3–2.0)	99.7 (99.6–99.8)	76 (59–104)

*Abbreviations: CI* confidence interval, *PPV* positive predictive values, *NPV* negative predictive value, *NNLP* number needed to lumbar puncture, *IMCI* integrated management of childhood infection

‘Or any of the above’ refers to the presence of ≥1 of the signs indicated in the preceding rows. This means that children represented on each row had the sign indicated on a particular row +/- any of the preceding signs. For example, 161 children had either bulging fontanel or neck stiffness (13 had both). 1023 children had ≥1 focal seizures, seizures outside the febrile convulsion age range, cyanosis, bulging fontanel, or neck stiffness (252 had focal seizures only while 771 had focal seizures and ≥1 of seizures outside the febrile convulsion age range, cyanosis, bulging fontanel, and neck stiffness [e.g. 76 had focal seizures which occurred outside the febrile convulsion age range; 1 had focal seizures which occurred outside the febrile convulsion age range and cyanosis])

^a^Bulging fontanel was only deemed present if the age was ≤18 months

#### IMCI

One or more IMCI signs were present in 4951 children, of whom 78 (1.6%) had meningitis compared with 20/7984 (0.3%) without IMCI signs: sensitivity 80% (95% CI 70–87), specificity 62% (95% CI 61–63), PPV 1.6% (95% CI 1.3–2.0), and NPV 100% (99%CI 99–100). Seventy-six children (95% CI 59–104) presenting with ≥1 IMCI signs would need to undergo an LP for each case of meningitis identified (Table 3).

#### Admission versus later LP

**Fig. 2:**
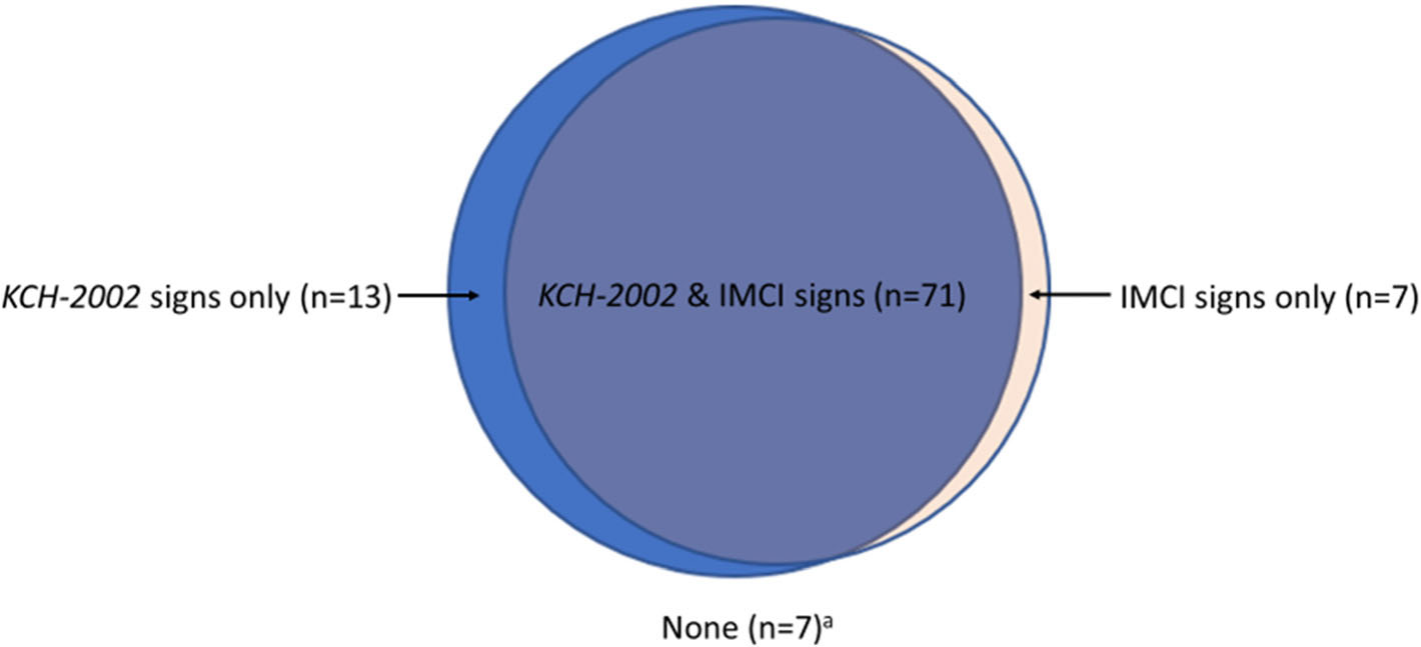
Clinical features of meningitis in 98 children with definite meningitis. Abbreviations: KCH-2002, previously identified signs at Kilifi Country Hospital; IMCI, Integrated Management of Childhood Illness. ^a^ History of fever with positive malaria smear (*n*=1), history of diarrhoea (*n*=2), history of vomiting (*n*=2), oedema (*n*=1), palmar pallor (*n*=1), severe acute malnutrition (*n*=2), died (*n*=2)

Thirty-three (34%) meningitis cases had their LP after admission, of which 6/33 (18%) and 8/33 (24%) were not identified by *KCH-2002* signs and IMCI signs, respectively, at admission. Seven (7.1%) meningitis cases were not identified by either *KCH-2002* signs or IMCI signs at admission (Fig. 2).

#### Sensitivity analysis

Excluding 565 who died before an LP (Group G) and including 51 cases with possible meningitis (Group D) as ‘meningitis’ gave similar results for *KCH-2002* and IMCI signs (Table S2). Fifty children with microbiologically confirmed meningitis fulfilled criteria as follows: 31 positive CSF cultures only (of which 23 had positive blood culture), 7 positive antigen tests only (of which 2 had positive blood culture), 5 positive microscopies (of which 2 had CSF leukocyte count >10), and 7 CSF leukocyte counts >10 cells/μL plus positive blood culture. For microbiologically confirmed meningitis, *KCH-*2002 signs had a sensitivity of 90% (95% CI 78–97) and specificity of 39% (95% CI 38–39). IMCI signs had a sensitivity of 76% (95% CI 62–87) and specificity of 63% (95% CI 62–64).

## Discussion

Misdiagnosis of bacterial meningitis based on clinical signs only may result in overtreatment with prolonged courses of antimicrobials, or undertreatment of missed cases [24], both contributing to mortality and selection of resistant organisms.

We studied a large cohort of hospitalised children to validate the clinical features of bacterial meningitis. Using the same definitions and inclusion criteria as in 2002, we observed a reduction in the prevalence of bacterial meningitis among paediatric admissions at our centre from 2% in 2001–2002 [11] to 0.8% in 2012–2016. There was also a decline in annual paediatric admissions and number of LPs done. However, we observed an increase in the prevalence of *KCH-2002* signs (55% in 2001–2002 vs 63% in 2012–2016, *P<0.001*) and a decrease in the prevalence of IMCI signs (42% in 2001–2002 vs 38% in 2012–2016, *P<0.001*) [11]. Although *S. pneumoniae* and *H. influenzae* remained the leading causes of bacterial meningitis, cases arising from these organisms declined over time (57 vs 20 pneumococcal, and 66 vs 8 *H. influenzae* cases, comparing 1994–1998 [25] to 2012-2016). These changes may be attributed to conjugate vaccination and herd immunity in older children. Our study excluded infants aged <60 days who typically have bacterial meningitis due to different pathogens [17], different clinical presentation [26], and alternative diagnoses such as birth asphyxia [27], and associated higher risk of neurological disability and mortality [17].

Clinical guidelines for limited-resource settings should comprise straightforward features, easily identifiable by clinicians [28]. Overall, we found that the clinical signs at admission had lower sensitivity and PPV in discriminating children with bacterial meningitis than in 2002 [11]. *KCH-2002* and IMCI signs did not statistically significantly differ in the proportions of meningitis cases missed (14% vs 20%, *P=0.258*), although numbers were limited for this comparison. Results did not appear to be altered by the exclusion of children who died before LP or using a narrower microbiological case definition.

History of fever was common with (90%) or without meningitis (68%) and nearly half of the LPs were done in children with malaria since signs overlap. The previous *KCH-2002* analysis found that exclusion of fever without malaria parasitaemia from the screening rule had lower sensitivity but higher specificity (sensitivity 79%, specificity 80%, PPV 8.0%) than when it was included (sensitivity 97%, specificity 44%, PPV 3.5%) [11]. The present analysis also shows that although the specificity of *KCH-2002* signs excluding fever without malaria parasitaemia has not changed, sensitivity was again markedly reduced (to 58% from 86%). Malaria parasitaemia has been shown to augment predictive models for bacterial meningitis [11, 29]; however, the significant morbidity and mortality associated with meningitis means a screening rule with higher sensitivity may be favourable despite lower specificity.

Although conjugate vaccination has resulted in a reduction in bacterial meningitis cases, antimicrobial resistance to penicillin [30] and chloramphenicol [31, 32] is reported. Ceftriaxone as a first-line treatment for bacterial meningitis has been associated with lower resistance rates, and reduction in mortality and neurological complications compared to chloramphenicol [32, 33]. Thus, clinical decision rules with optimal performance in predicting bacterial meningitis contribute to antimicrobial stewardship by guiding initiation of treatment and minimising selection of resistant microorganisms.

### Limitations

An inescapable limitation is that a selective group of children underwent an LP based on clinical suspicion at admission or later during admission. It is possible that a number of bacterial meningitis cases may have been missed due to apparent recovery and discharge. However, we believe that the higher than usual clinical staffing, training oversight, and availability of laboratory resources due to the presence of the research programme helped limit the chances of missed meningitis cases. Although performing LPs in all children is diagnostically optimal and would provide an understanding of the true prevalence of meningitis, this is not possible due to the risks involved and would not be ethically justified [22]. Our dataset may not be perfect, but it addresses research gaps in similar settings. Of 2602 LPs done, 1026 (39%) were performed after admission; 33/98 (34%) meningitis cases were diagnosed after admission, underscoring the importance of daily clinical reviews following standard guidelines. Our assumption of true negatives in children who did not develop signs suggestive of meningitis during hospitalisation and were discharged home alive is valid. The highest proportion of children having an LP was in those aged 1–5 years. *KCH-2002* signs were most frequent among children aged <1 year, fewer LPs done in this age group may be attributed to early deaths or more LPs being deferred due to contraindications since most deaths occurred in young infants (7.4%, 4.3%, and 4.4% deaths in children aged <1, 1–5, and ≥5 years, respectively, *P<0.001*). However, age bias in LPs may have affected our findings. Importantly, our aim was to inform clinical guidelines for empiric treatment and indications for LP rather than describe the epidemiology of meningitis for which post-mortem LPs would have been necessary.

Molecular tests for bacterial and viral causes were not routinely done, potentially missing true bacterial meningitis cases and falsely including viral meningitis cases. Although differential leukocyte count was done in some CSF samples, it was not included in our standard definition of meningitis. Polymorphonuclear cell predominance can occur in both bacterial and aseptic meningitis [34]. We lacked data on pre-hospital antibiotic exposure which may be common and has been shown to alter CSF leukocyte count and biochemical profile and impede detection of bacterial pathogens [5, 35]. Diagnostic delay may decrease survival [36] and increase neurological sequelae in Hib meningitis [37] and may be more of a problem in settings without advanced diagnostic resources such as CSF polymerase chain reaction (PCR) [38].

Low LP rates reported in settings like ours have raised concerns regarding missing meningitis cases [6, 7]. Knowing that a large number of LPs is needed in order to diagnose each case of bacterial meningitis is important in this regard. The *KCH-2002* or IMCI signs at admission suggest an LP may be needed in ~40 to 60% of children presenting to the hospital with these signs to achieve >80% sensitivity. There are no studies evaluating the additional discriminatory value of a structured repeated evaluation of signs that develop later during admission, or of biomarkers in this context. Although traumatic LPs are common and may complicate CSF leukocyte interpretation, adjustment of CSF leukocyte count has been shown to lack additional value in predicting meningitis [39]. In our study, only 5 children with CSF RBC ≥500 cells/μL met our laboratory meningitis criteria. Our results provided important guidance for performing LPs in LMICs settings where there is a paucity of comprehensive data on this important question.

## Conclusions

Bacterial meningitis is an uncommon but important diagnosis in children. Declining incidence is welcome but identifying children with meningitis has become more difficult. Clinicians and policymakers should be aware of the number of LPs or empirical treatments needed for each case of bacterial meningitis to be identified, and this may vary with malaria endemicity. The IMCI criteria offer a balance between the more specific *KCH-2002* signs (impaired consciousness or any one of bulging fontanel, neck stiffness, cyanosis, seizures outside 6 months to 6 years, or focal seizures) and non-malarial fever. While the IMCI criteria will continue to be used, the number of LPs needed to identify a single case of bacterial meningitis has increased 3-fold from 24 to 76. Clinicians should continue to have a high index of suspicion while assessing children during daily reviews. Support to establish accurate CSF cell counting, Gram stain, and glucose measurement as a minimum in resource-poor settings to optimise antimicrobial treatment is essential to providing effective inpatient paediatric services.

## Supplementary Information

**Additional file 1: Table S1.** Comparison of annual admissions, lumbar punctures and meningitis cases during our study period and our previous analysis. **Table S2.** Sensitivity Analysis of Potential Screening Criteria at Admission for Meningitis.

## Data Availability

The dataset used and analysed during the current study is available from the KWTRP Data Governance Committee (DGC) on reasonable request (dgc@kemri-wellcome.org), ensuring the protection of the privacy, rights and interests of research participants and primary researchers, and upholding transparency and accountability. KWTRP is the custodian of the data used in this analysis, and the KWTRP DGC oversees the internal data repository.

## Abbreviations

LMICs: Low- and middle-income countries
CSF: Cerebrospinal fluid
LP: Lumbar puncture
WHO: World Health Organization
Hib: Haemophilus influenzae type b
IMCI: Integrated Management of Childhood Illness
KCH: Kilifi County Hospital
PPV: Positive predictive value
NPV: Negative predictive value
