# Supplementary material for: Clinical features of bacterial meningitis among hospitalised children in Kenya
Source: BMC Med. 2021 Jun 4;19:122. doi: 10.1186/s12916-021-01998-3 (PMC8176744; doi:10.1186/s12916-021-01998-3)
Supplement: Supplementary file 1 — Additional file 1: Table S1. Comparison of annual admissions, lumbar punctures and meningitis cases during our study period and our previous analysis. Table S2. Sensitivity Analysis of Potential Screening Criteria at Admission for Meningitis. [file 12916_2021_1998_MOESM1_ESM.docx]

Additional file 1

Clinical features of bacterial meningitis among hospitalized children in Kenya

Christina W. Obiero MBChB, MPH^1,2^, Neema Mturi MRCP^1^, Salim Mwarumba MSc^3^, Moses Ngari DPhil^1,4^, Charles R. Newton MD FRCPCH^1,5^, Michaël Boele van Hensbroek PhD^2^, and James A. Berkley FRCPCH FMedSci ^1,4,6^

^1^ Clinical Research Department, KEMRI-Wellcome Trust Research Programme, Kilifi, Kenya

^2^ Department of Global Health, University of Amsterdam Faculty of Medicine, Amsterdam, Noord-Holland, The Netherlands

^3^ Department of Microbiology, KEMRI-Wellcome Trust Research Programme, Kilifi, Kenya

^4^ The Childhood Acute Illness and Nutrition (CHAIN) Network, Nairobi, Kenya

^5^ Department of Psychiatry, University of Oxford, Oxford, UK.

^6^ Centre for Tropical Medicine & Global Health, Nuffield Department of Medicine, University of Oxford, Oxford, UK

## *Table S1. Comparison of annual admissions, lumbar punctures and meningitis cases during our study period and our previous analysis*

| **Study period** | **2012-2016** | **2001-2002** |
| --- | --- | --- |
| Admissions/year | 2,597 | 4,616 |
| Lumbar punctures/year | 520 | 999 |
| Meningitis cases/year | 20 | 91 |
| Data are N | | |

## *Table S2. Sensitivity Analysis of Potential Screening Criteria at Admission for Meningitis*

| **Screening Criteria** | **Meningitis (n=98) vs no meningitis (n= 12,272)^a^** | **Meningitis (plus possible meningitis) (n=149) vs no meningitis (n=12,837)^b^** | **Meningitis (plus possible meningitis) (n=149) vs no meningitis (n=12,272)^c^** | **Microbiologically confirmed meningitis^d^ (n=50) vs no meningitis (n=12,371)^c^** |
| --- | --- | --- | --- | --- |
| Bulging fontanel^e^ or neck stiffness |  |  |  |  |
| No. with criteria | 147 | 166 | 152 | 152 |
| No. with meningitis | 23 | 28 | 28 | 14 |
| Sensitivity (95% CI) | 23.5 (15.5-33.1) | 18.8 (12.9-26.0) | 18.8 (12.9-26.0) | 28.0 (16.2-42.5) |
| Specificity (95% CI) | 99.0 (98.8-99.2) | 98.9 (98.7-99.1) | 99.0 (98.8-99.2) | 98.9 (98.7-99.1) |
| PPV (95% CI) | 15.6 (10.2-22.5) | 16.9 (11.5-23.4) | 18.4 (12.6-25.5) | 9.2 (5.1-15.0) |
| NPV (95% CI) | 99.4 (99.2-99.5) | 99.1 (98.9-99.2) | 99.0 (98.8-99.2) | 99.7 (99.6-99.8) |
| NNLP (95% CI) | 7 (5-11) | 6 (5-10) | 6 (4-9) | 11 (7-23) |
| Cyanosis or any of the above |  |  |  |  |
| No. with criteria | 178 | 212 | 183 | 183 |
| No. with meningitis | 23 | 28 | 28 | 14 |
| Sensitivity (95% CI) | 23.5 (15.5-33.1) | 18.8 (12.9-26.0) | 18.8 (12.9-26.0) | 28.0 (16.2-42.5) |
| Specificity (95% CI) | 98.7 (98.5-98.9) | 98.6 (98.3-98.8) | 98.7 (98.5-98.9) | 98.6 (98.4-98.8) |
| PPV (95% CI) | 12.9 (8.4-18.8) | 13.2 (9.0-18.5) | 15.3 (10.4-21.3) | 7.7 (4.3-12.5) |
| NPV (95% CI) | 99.4 (99.2-99.5) | 99.1 (98.9-99.2) | 99.0 (98.8-99.2) | 99.7 (99.6-99.8) |
| NNLP (95% CI) | 8 (6-14) | 8 (6-13) | 7 (5-11) | 14 (9-29) |
| Seizures outside 6 mo to 6 y or any of the above |  |  |  |  |
| No. with criteria | 715 | 787 | 731 | 731 |
| No. with meningitis | 33 | 49 | 49 | 21 |
| Sensitivity (95% CI) | 33.7 (24.4-43.9) | 32.9 (25.4-41.0) | 32.9 (25.4-41.0) | 42.0 (28.2-56.8) |
| Specificity (95% CI) | 94.4 (94.0-94.8) | 94.3 (93.8-94.6) | 94.4 (94.0-94.8) | 94.3 (93.8-94.7) |
| PPV (95% CI) | 4.6 (3.2-6.4) | 6.2 (4.6-8.2) | 6.7 (5.0-8.8) | 2.9 (1.8-4.4) |
| NPV (95% CI) | 99.4 (99.3-99.6) | 99.2 (99.0-99.3) | 99.1 (99.0-99.3) | 99.8 (99.6-99.8) |
| NNLP (95% CI) | 25 (18-40) | 19 (14-27) | 17 (13-25) | 38 (26-71) |
| Focal seizures or any of the above |  |  |  |  |
| No. with criteria | 954 | 1,043 | 974 | 974 |
| No. with meningitis | 39 | 59 | 59 | 23 |
| Sensitivity (95% CI) | 39.8 (30.0-50.2) | 39.6 (31.7-47.9) | 39.6 (31.7-47.9) | 46.0 (31.8-60.7) |
| Specificity (95% CI) | 92.5 (92.1-93.0) | 92.3 (91.9-92.8) | 92.5 (92.1-93.0) | 92.3 (91.8-92.8) |
| PPV (95% CI) | 4.1 (2.9-5.6) | 5.7 (4.3-7.2) | 6.1 (4.6-7.7) | 2.4 (1.5-3.5) |
| NPV (95% CI) | 99.5 (99.3-99.6) | 99.2 (99.1-99.4) | 99.2 (99.0-99.4) | 99.8 (99.7-99.8) |
| NNLP (95% CI) | 28 (21-43) | 20 (16-29) | 19 (15-27) | 47 (32-86) |
| Impaired consciousness or any of the above |  |  |  |  |
| No. with criteria | 2,354 | 2,690 | 2,385 | 2,385 |
| No. with meningitis | 57 | 88 | 88 | 31 |
| Sensitivity (95% CI) | 58.2 (47.8-68.1) | 59.1 (50.7-67.0) | 59.1 (50.7-67.0) | 62.0 (47.2-75.3) |
| Specificity (95% CI) | 81.3 (80.6-82.0) | 79.7 (79.0-80.4) | 81.3 (80.6-82.0) | 81.0 (80.3-81.7) |
| PPV (95% CI) | 2.4 (1.8-3.1) | 3.3 (2.6-4.0) | 3.7 (3.0-4.5) | 1.3 (0.9-1.8) |
| NPV (95% CI) | 99.6 (99.4-99.7) | 99.4 (99.2-99.5) | 99.4 (99.2-99.5) | 99.8 (99.7-99.9) |
| NNLP (95% CI) | 50 (38-73) | 37 (30-50) | 32 (26-43) | 90 (64-154) |
| Fever without malaria parasitaemia or any of the above |  |  |  |  |
| No. with criteria | 7,614 | 8,142 | 7,657 | 7,657 |
| No. with meningitis | 84 | 127 | 127 | 45 |
| Sensitivity (95% CI) | 85.7 (77.2-92.0) | 85.2 (78.5-90.5) | 85.2 (78.5-90.5) | 90.0 (78.2-96.7) |
| Specificity (95% CI) | 38.6 (37.8-39.5) | 37.6 (36.7-38.4) | 38.6 (37.8-39.5) | 38.5 (37.6-39.3) |
| PPV (95% CI) | 1.1 (0.9-1.4) | 1.6 (1.3-1.9) | 1.7 (1.4-2.0) | 0.6 (0.4-0.8) |
| NPV (95% CI) | 99.7 (99.5-99.8) | 99.5 (99.3-99.7) | 99.5 (99.3-99.7) | 99.9 (99.8-100.0) |
| NNLP (95% CI) | 124 (92-189) | 90 (70-129) | 84 (65-117) | 207 (148-347) |
| IMCI referral criteria: neck stiffness, lethargy, impaired consciousness, or seizures |  |  |  |  |
| No. with criteria | 4,592 | 4,994 | 4,635 | 4,635 |
| No. with meningitis | 78 | 121 | 121 | 38 |
| Sensitivity (95% CI) | 79.6 (70.3-87.1) | 81.2 (74.0-87.1) | 81.2 (74.0-87.1) | 76.0 (61.8-86.9) |
| Specificity (95% CI) | 63.2 (62.4-64.1) | 62.0 (61.2-62.9) | 63.2 (62.4-64.1) | 62.8 (62.0-63.7) |
| PPV (95% CI) | 1.7 (1.3-2.1) | 2.4 (2.0-2.9) | 2.6 (2.2-3.1) | 0.8 (0.6-1.1) |
| NPV (95% CI) | 99.7 (99.6-99.8) | 99.6 (99.5-99.8) | 99.6 (99.5-99.8) | 99.8 (99.7-99.9) |
| NNLP (95% CI) | 69 (55-95) | 48 (40-61) | 44 (37-56) | 150 (106-255) |
| Abbreviations: CI, confidence interval; PPV, positive predictive values; NPV, negative predictive value; NNLP, number needed to lumbar puncture; IMCI, integrated management of childhood infection.  ‘Or any of the above’ refers to presence of ≥1 of the signs indicated in the preceding rows. This means that children represented on each row had the sign indicated on a particular row +/- any of the preceding signs.  ^a^Excludes children with possible meningitis (Group D, n=51) and children who died before an LP (Group G, n=565)  ^b^Includes children who died before an LP (Group G, n=565)  ^c^Excludes children who died before an LP (Group G, n=565)  ^d^Positive CSF culture, antigen test, microscopy, or CSF leukocytes >10/ µL plus positive blood culture  ^e^Bulging fontanel was only deemed present if the age was ≤18 months | | | | |
